# Identification of Cancer Dysfunctional Subpathways by Integrating DNA Methylation, Copy Number Variation, and Gene-Expression Data

**DOI:** 10.3389/fgene.2019.00441

**Published:** 2019-05-15

**Authors:** Siyao Liu, Baotong Zheng, Yuqi Sheng, Qingfei Kong, Ying Jiang, Yang Yang, Xudong Han, Liang Cheng, Yunpeng Zhang, Junwei Han

**Affiliations:** ^1^College of Bioinformatics Science and Technology, Harbin Medical University, Harbin, China; ^2^College of Basic Medical Science, Harbin Medical University, Harbin, China; ^3^College of Basic Medical Science, Heilongjiang University of Chinese Medicine, Harbin, China

**Keywords:** multi-omics data, copy number variation, DNA methylation, subpathway activity, pathway topological information

## Abstract

A subpathway is defined as the local region of a biological pathway with specific biological functions. With the generation of large-scale sequencing data, there are more opportunities to study the molecular mechanisms of cancer development. It is necessary to investigate the potential impact of DNA methylation, copy number variation (CNV), and gene-expression changes in the molecular states of oncogenic dysfunctional subpathways. We propose a novel method, Identification of Cancer Dysfunctional Subpathways (ICDS), by integrating multi-omics data and pathway topological information to identify dysfunctional subpathways. We first calculated gene-risk scores by integrating the three following types of data: DNA methylation, CNV, and gene expression. Second, we performed a greedy search algorithm to identify the key dysfunctional subpathways within pathways for which the discriminative scores were locally maximal. Finally, a permutation test was used to calculate the statistical significance level for these key dysfunctional subpathways. We validated the effectiveness of ICDS in identifying dysregulated subpathways using datasets from liver hepatocellular carcinoma (LIHC), head-neck squamous cell carcinoma (HNSC), cervical squamous cell carcinoma, and endocervical adenocarcinoma. We further compared ICDS with methods that performed the same subpathway identification algorithm but only considered DNA methylation, CNV, or gene expression (defined as ICDS_M, ICDS_CNV, or ICDS_G, respectively). With these analyses, we confirmed that ICDS better identified cancer-associated subpathways than the three other methods, which only considered one type of data. Our ICDS method has been implemented as a freely available R-based tool (https://cran.r-project.org/web/packages/ICDS).

## Introduction

Cancer is a complex disease involving multiple biological processes and multiple factors, including genomic, epigenomic, and transcriptomic aberrations associated with cancer formation and development (Forozan et al., 2000; Zhang et al., 2012). Identifying molecular markers of cancer is a major challenge and can effectively clarify diagnosis and treatment. With the development of high-throughput sequencing technology, it is possible to understand the pathogenic mechanisms of cancer at the molecular level (Wang et al., 2014; Liu and Xu, 2015; Zhang et al., 2017). Large-scale cancer genomics projects, such as the Cancer Genome Atlas (TCGA) (Giordano, 2014), provide multi-omics profiles from a large number of patient samples from many cancer types. This may provide a basis for the systematic understanding of the development of cancer. However, both copy number variation (CNV) and DNA methylation changes may affect gene expression, and integration of these data may enhance essential gene characterization in cancer progression (Kim et al., 2010; Xu et al., 2010). Many studies have shown that the use of multi-omics data for integrated analysis helps us to understand the pathogenic mechanisms of cancer. For example, Xu et al. (2010) have shown that the correlation between gene expression and CNV has biological effects on carcinogenesis and cancer progression. Additionally, Zhang et al. (2013) has classified the prognosis of patients with different subtypes of ovarian cancer by integrating four types of molecular data related to gene expression. In view of these works, our goal is to explore the multi-layered genetic and epigenetic regulatory mechanisms of cancer.

Biological pathways are models containing structural information between genes, such as interactions, regulation, modifications, and binding properties. In addition, genes in the same pathway usually coordinately achieve a particular function. With the appearance of some traditional pathway-analysis tools, such as GSEA (Subramanian et al., 2005) and SPIA (Tarca et al., 2009), the pathway-based approach has become the first choice for complex disease analysis to facilitate biological insights. Existing biological-pathway databases provide pathway topological information, such as with the Kyoto Encyclopedia of Genes and Genomes (KEGG) (Wixon and Kell, 2000), which is being updated to suit the needs for practical applications and act a systematic reference knowledge database to understand the metabolism and other cellular processes. Recently, the KEGG pathway database has become one of most widely used resource for biological function annotation (Kanehisa et al., 2017).

Based on pathway topological information, the subpathway concept was proposed in our previous study in which we confirmed that key subpathways – rather than entire pathways – were more suitable for explaining the etiology of diseases (Li et al., 2009, 2013). Subpathways contain fewer components, which enables a more accurate interpretation of the biological function of the disturbance, for the future study of precision medicine. Subpathway-GM (Li et al., 2013) was proposed to identify disease-relevant subpathways by integrating information across genes, metabolites, and pathway structural information within a given pathway; using this, 16 statistically significant subpathways were identified as associated with metastatic prostate cancer. SubpathwayMiner (Li et al., 2009) uses a subgraph-mining method to find subpathways where all of the genes have highly similar functions; this method identified36 dysfunctional subpathways – enriched by differentially expressed genes – as associated with the initiation or progression of lung cancer. Recently, some other methods have been developed to identify subpathways from pathway topology. One example is MIDAS (Lee et al., 2017), which determines condition-specific subpathways and fully utilizes quantitative gene-expression data and network-centrality information across multiple phenotypes. Moreover, the following subpathway-activity measurement tools have been designed to identify activated subpathways between two phenotypes: PATHOME (pathway and transcriptome information) (Nam et al., 2014), TEAK (Topology Enrichment Analysis frameworK) (Judeh et al., 2013), and MinePath (Mining for Phenotype Differential Sub-paths in Molecular Pathways) (Koumakis et al., 2016). Moreover, there is also some other methods proposed network-based analysis to discover *de novo* pathway. For instance, *de novo* pathway enrichment extracted sub-networks enriched in biological entities active by combining experimental data with a large-scale interaction network (Batra et al., 2017). These subpathway-analysis methods mainly identify dysfunctional subpathways only by comparing the expression levels of their involved genes between tumor and normal tissues. In this way, other genetic characterizations of genes, such as CNVs and DNA methylation, are ignored. However, both DNA methylation and CNVs in cancer genomes frequently perturb the expression levels of affected genes and, thus, disrupt pathways controlling normal growth. It is therefore necessary to integrate gene expression information and other genetic information, such as DNA methylation and CNVs, to identify dysfunctional subpathways.

In this study, we propose a novel method, termed Identification of Cancer Dysfunctional Subpathways (ICDS), to identify dysfunctional subpathways by integrating multi-omics data and pathway topological information. In ICDS, the first step is to calculate gene-risk scores to evaluate the contribution of genes to cancer states by considering the following three molecular characterizations: DNA methylation, CNV, and gene expression. In the second step, we convert the KEGG pathway into an undirected-pathway network with genes as nodes and biological relationships as edges, and use a greedy search algorithm to search for candidate dysfunctional subpathways within the pathways for which the discriminative scores are locally maximal. Finally, a perturbation test is used to calculate statistical significance for these dysfunctional subpathways. We applied the ICDS method to liver hepatocellular carcinoma (LIHC), head-neck squamous cell carcinoma (HNSC), and cervical squamous cell carcinoma and endocervical adenocarcinoma (CESC) datasets, and compared our results with three analytical methods that only used DNA methylation, CNV, or gene expression to calculate subpathway-activity scores (defined as ICDS_G, ICDS_CNV, ICDS_M, respectively). Through these analyses, we confirmed that ICDS could better identify cancer-associated subpathways compared to the other three methods.

## Materials and Methods

### Datasets

The datasets containing gene expression, CNV, and DNA methylation information were collected from the TCGA website^1^. We downloaded TCGA RNA-seq level-3 data, which were processed and normalized and used the Reads Per Kilobase per Million mapped reads (RPKM) values for the gene-expression levels. Finally, there were 19,754 genes used in 424 LIHC, 546 HNSC, and 309 CESC samples. CNV profiling was estimated using the GISTIC2 method (Mermel et al., 2011), and was annotated to genes using the UCSC cgData HUGO probeMap. For example, the LIHC dataset contains CNVs in 24,776 genes from 373 cancer samples. In this study, we further filtered 364 LIHC samples with matched gene-expression profiles.

We downloaded TCGA level-3 Illumina HumanMethylation450 Bead Array data for DNA methylation. The LIHC DNA methylation level-3 dataset contain β-values for 20,105 genes from 429 samples, which included 50 normal samples and 379 lung-cancer samples. The β-values are calculated by *M*/(*M*+*U*+100) with a range from 0 to 1, in which *M* is methylated allele frequencies and *U* is unmethylated allele frequencies. Overall, higher β-values indicate higher methylation. For three datasets, we removed genes with values of zero in more than 80% of the samples. In this paper, we also use the data from HNSC and CESC samples, which were processed using the above procedure. Detailed data information is shown in Supplementary Table S1.

The KEGG pathway database contains experimentally verified pathway structural information (e.g., interactions, regulation, modifications, and binding between genes). We collected 294 KEGG biological pathways, and each pathway was converted to an undirected network with genes as nodes and biological relationships as edges on the basis of pathway structural information using the “iSubpathwayMiner” system (Li et al., 2009, 2013).

### Calculated Gene Risk Score in Cancer

There are many factors influencing tumorigenesis, such as gene expression, CNV and DNA methylation. For each gene, we calculated its risk score in cancer by considering the following three types of genetic molecular features: gene expression, CNV, and DNA methylation. With the above data, we used the Student’s *t*-test (Hogben, 1964) to calculate the adjusted *p*-value for differential expression level and differential methylation level of each gene in the tumor and normal samples (denoted by *p_gene_* and *p_methy_*). According to results of GISTIC2 analysis, the sample was then divided into a copy-number-variated group and an un-variated group for each gene, and then the differential expression level of the gene in the two groups was calculated by Student’s *t*-test (denoted by *p_cnv_*). It is difficult to define the quantitative relationship and relative degree of each factor’s influence on tumorigenesis, so we assume that gene expression, CNV, and DNA Methylation equally contribute to the cancer development. The gene risk score (*RS*) was calculated by integrating the above three *p*-values with Fisher’s combined probability test. This method computed a combined statistic *S* from the adjusted *p*-values obtained from the three individual datasets as shown in Equation (1). Usually, the statistic *S* followed a χ^2^ distribution with 2*k* degrees of freedom, and we then calculated the null hypothesis *p*-value of the statistic *S*. Finally, we converted the *p*-value to a *z*-score according to the inverse-normal cumulative-distribution function (CDF), and the z-score was taken as the *RS* of each gene in cancer.

(1) $$S=-2\log\prod_{m}p_{m},\;\;\;m=gene,\;cnv,\;methy$$

### Calculated Subpathway-Activity Score

Previous studies have confirmed that subpathways can provide more detailed biological information than whole pathways. In this study, we proposed a novel method to combine gene-risk score with pathway topological structure to infer subpathway activities. The *RS* of genes were obtained by the above method, considering gene expression, CNV and methylation. For a KEGG pathway, we performed a greedy algorithm to search for dysfunctional subpathways within the pathways for which the discriminative scores were locally maximal. Specifically, the search algorithm started from a seed gene *i* which had a significantly high risk score (*p* < 0.001) and expanded iteratively, after which it selected one of the neighbors of the seed gene to form the current subpathway. For a subpathway *k*, the activity score (*AS_k_*) was the average of the *RS* of the member genes in the subpathway, calculated by Equation (2):

(2) $$AS_{k}=\sum_{i}\frac{RS_{i}}{\sqrt{n}}$$

In Equation (2), *i* is the index of the gene in the subpathway *k*, while *n* is the number of genes involved in the subpathway. At each iteration, the algorithm adopted a gene from the neighbors of genes in the current subpathway, which produced maximal increases between *AS_k+1_* and *AS_k_*. The search algorithm will stops when no additional gene increases in the score *AS_k+1_* over (1+*r*) *AS_k_* or the distance in the current subpathway between any two nodes is greater than 3 in order to keep the search locally. The improvement rate *r* is chosen to avoid too large subpathway region, resulting in the addition of redundant weak information. The parameter *r* = 0.05 has been demonstrated to be appropriate in the greedy heuristic algorithm applied in the biological network (Chuang et al., 2007). When the Jaccard index between each pair of subpathways in the same pathway was more than 0.6, they were combined, which ensured that the subpathways we found in our method contained more information and reduce redundancy. Furthermore, we only considered subpathways with more than five genes and less than 100 genes, to avoid overly narrow or broad functional subpathways.

### Significance Test of the Subpathway

We provided two statistical test methods for each candidate subpathway, of which one was a whole gene-based perturbation, and the other was a local-gene perturbation in a particular pathway. Users can choose the test method that they prefer. The first test perturbs the gene labels on the entire gene list in the pathway networks, and recalculates the activity score of the subpathway, denoted as *AS_k_perm1_*. This test was used to test the correlation between real subpathways and disease phenotype. In this study, we performed 10,000 perturbations for this test and calculated statistically significant *p*-value = *M*/*N*, in which *M* is the number of *AS_k_perm1_* greater than the real subpathway score *AS_k_*, and *N* is the number of perturbations. In addition, the second test perturbed the gene names in the pathway to which the subpathway belonged, and recalculated the activity score of the subpathway, denoted as *AS_k_perm2_*. This test was used to test the correlation between real subpathways and pathway structure. We also performed 10,000 perturbations and the score of each real *AS_k_* was indexed on the null distribution of all *AS_k_perm2_* whose *p*-values could be evaluated. The *p*-values were adjusted using the false discovery rate (FDR) method proposed by Benjamini and Hochberg to correct for multiple comparisons (Benjamini et al., 2001). In this study, both FDR at 0.001 was used as the subpathway-significance threshold. We have implemented ICDS as an R-based package that is publicly available on CRAN^2^.

## Results

### Analyses of Hepatocellular Carcinoma Data

**FIGURE 1:**
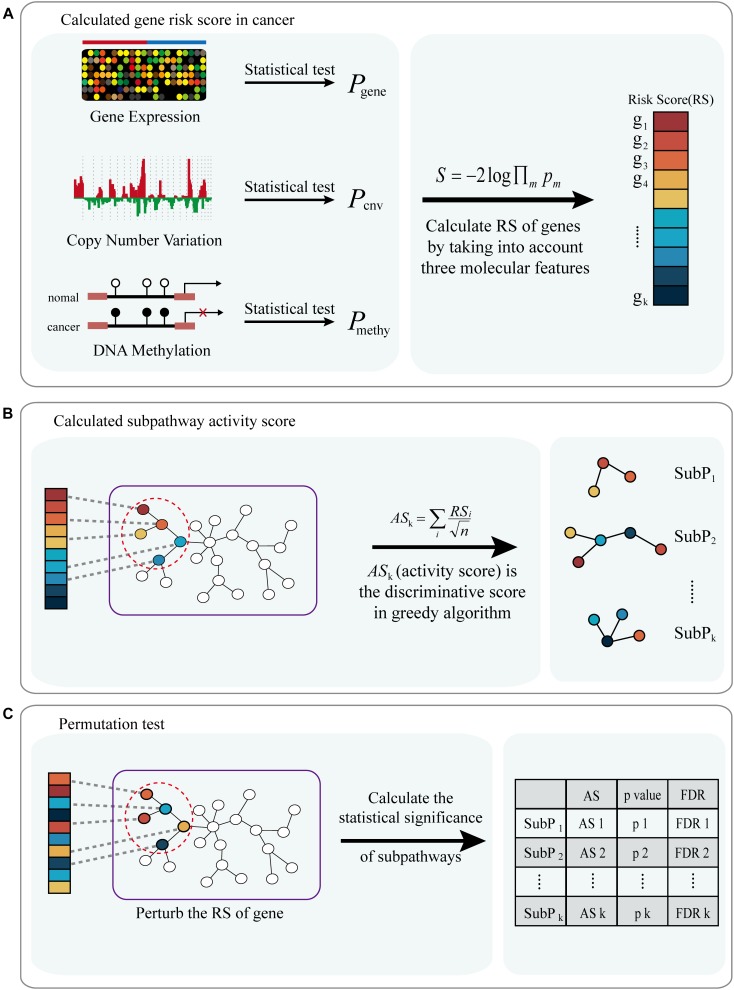
Flow diagram of ICDS methodology. **(A)** Calculated risk score of genes (*RS*) in cancer by considering three types of genetic molecular features: gene expression, CNV and DNA methylation. **(B)** Combine gen-risk score with pathway topological structure to infer the subpathway activity score (AS); subpathways with discriminative activity score in cancer were identified via a greedy search algorithm. **(C)** A permutation test is performed on the risk score of genes, and pathways are prioritized by FDR after permutation tests.

A workflow diagram of the ICDS is shown in Figure 1. We first applied ICDS to identify dysfunctional subpathways in LIHC. The LIHC dataset was obtained from TCGA, and its detailed information is shown in Supplementary Table S1. In the LIHC dataset, we calculated the risk score of 16,207 genes by considering the following three types of genetic molecular features: gene expression, CNV, and DNA methylation. We set the genes with *p* < 0.001 (derived from the combined statistic S) as the seed genes in the pathway network for the subpathway search algorithm (see Materials and Methods). Subpathways were selected which satisfied two permutation tests with FDR1 < 0.001 and FDR2 < 0.001 out of the 10,000 permutations. ICDS identified 19 dysfunctional subpathways associated with LIHC, belonging to 12 entire pathways (Table 1 and Supplementary Table S2), of which up to nine were reported to be associated with tumor occurrence, development, and metastasis.

**Table 1:** Subpathways identified by ICDS with FDR < 0.001 in the LIHC dataset.

SubpathID	Pathway	Size^∗^	FDR1	FDR2	ICDS-G	ICDS-CNV	ICDS-M
path:00230_1	Purine metabolism	61	<E-11	9.13E-11	√		
path:00240_1	Pyrimidine metabolism	51	<E-11	1.76E-07	√		
path:04380_1	Osteoclast differentiation	13	<E-11	3.29E-06			√
path:00830_1	Retinol metabolism	23	<E-11	3.29E-06			
path:04062_1	Chemokine signaling pathway	24	<E-11	3.46E-06			
path:04510_10	Focal adhesion	8	<E-11	3.46E-06			
path:04152_1	AMPK signaling pathway	24	<E-11	6.34E-06	√		
path:05166_1	HTLV-I infection	16	<E-11	6.34E-06			
path:04062_4	Chemokine signaling pathway	9	<E-11	9.45E-06			
path:00240_3	Pyrimidine metabolism	7	<E-11	1.23E-05			
path:04062_7	Chemokine signaling pathway	10	<E-11	1.31E-05			
path:04110_10	Cell cycle	8	<E-11	2.10E-05			
path:04110_11	Cell cycle	9	<E-11	3.13E-05			
path:04630_4	Jak-STAT signaling pathway	5	<E-11	3.43E-05			
path:00240_2	Pyrimidine metabolism	7	<E-11	3.75E-05			
path:00240_4	Pyrimidine metabolism	8	<E-11	6.61E-05			
path:00230_4	Purine metabolism	10	<E-11	1.10E-04			
path:04110_1	Cell cycle	25	<E-11	1.85E-04			
path:04114_1	Oocyte meiosis	28	<E-11	9.42E-04			


*^∗^The number of genes in the subpathway.*

The most significant subpathway was path 00230_1 in purine metabolism, which contained 61 genes. Some studies have confirmed that the purine-metabolism pathway is highly correlated with the occurrence and metastasis of liver cancer. In multiple cancer cells, a marked imbalance in the enzymic pattern of purine metabolism is linked with transformation or progression, such as in kidney, liver, and colon carcinomas (Weber, 1983). The subregion corresponding to the subpathway included 61 genes (Supplementary Figure S1A), such as adenosine monophosphate deaminase 1 (AMPD1) and adenosine kinase (ADK), which are important enzymes involved in purine metabolism. ADK plays a significant role in affecting apoptosis and may become a target for the treatment of cancer (Dzeja et al., 1998). More evidence is mounting regarding the direct relationship between defects in ADK and AMP metabolic signaling (e.g., AMPD) and human diseases (Pavlova and Thompson, 2016), which is a set of collaborative interactions that converts adenosine monophosphate (AMP) to inosine monophosphate (IMP) as part of the process of the purine nucleotide cycle. Compared with normal hepatocytes, the levels of ADK and AMPD1 in LIHC cells were significantly different in expression and methylation (*p_gene_* = 6.58e-05 of ADK and *p_gene_* = 0.0042 of AMPD1; *p_methy_* = 1.05e-05 of ADK and *p_methy_* = 9.48e-12 of AMPD1) (Supplementary Figure S1B). The abnormality of ADK and AMPD1 changes the metabolic homeostasis of cells and promotes the progression of cancer cells (Pedley and Benkovic, 2017).

**FIGURE 2:**
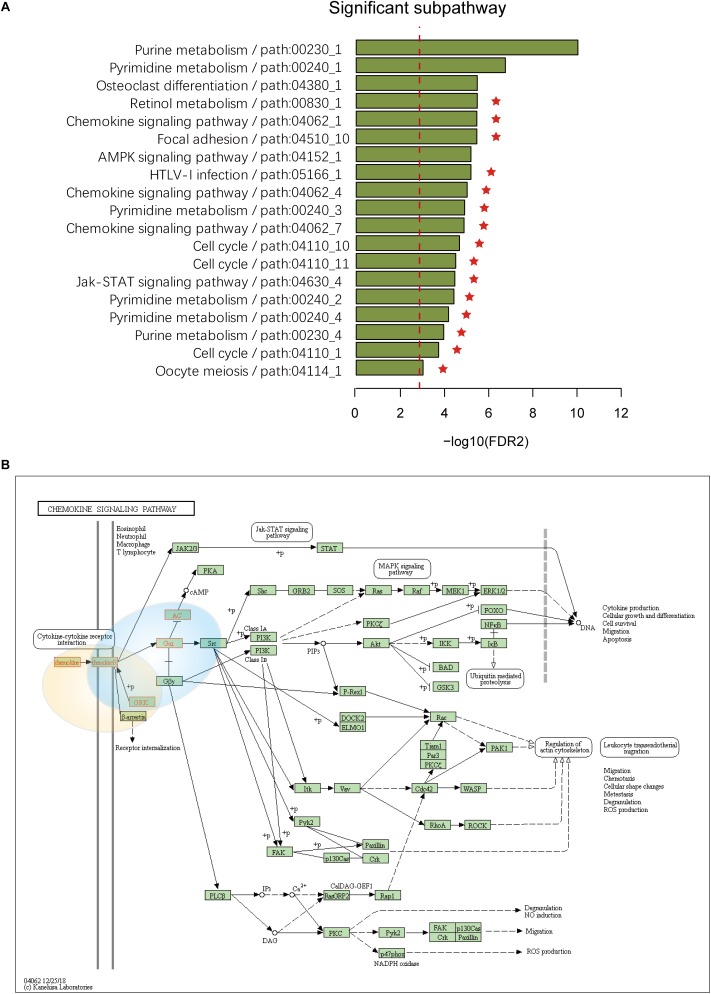
**(A)** Subpathways identified by ICDS with FDR < 0.001 in the LIHC dataset. The y-axis represents significant subpathways sorted by FDR2, while the x-axis represents the –log transformed FDR2. Compared to the three methods (ICDS-G, ICDS-CNV and ICDS-M), the subpathways exclusively identified by the ICDS method are marked with red asterisks. **(B)** Annotation of genes in subpathway path:04062_1 and path:04062_4 to the original chemokine signaling pathway in KEGG. Genes are marked with red, and the light-yellow circle corresponds to subpathway path:04062_1 and the blue circle to subpathway path:04062_4.

**FIGURE 3:**
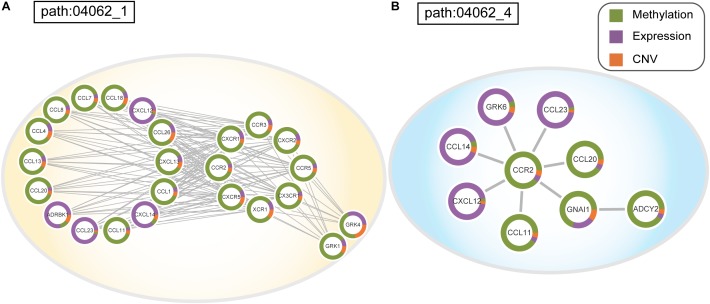
**(A)** Dysfunctional subpathway (path:04062_1) of chemokine signaling pathway in LIHC. **(B)** Dysfunctional subpathway (path:04062_4) of chemokine signaling pathway in LIHC. The vertex in the subnetwork represents a gene, and green and purple colors in the vertex represent the proportion of the gene’s differential expression scores and differential methylation scores between cancer samples and normal samples; orange colors represent the proportion of influence of CNV on gene expression.

To assess the effectiveness of ICDS, we compared our results in LIHC with three other analytical methods in which we calculated the RS of genes by considering only one of the following types of data: gene expression, CNV, or DNA methylation (defined as ICDS-G, ICDS-CNV, or ICDS-M, respectively). Next, we used the same procedure as above to find significant subpathways and used the same parameter settings. Using the methods of ICDS-G and ICDS-M, we obtained three and one significant subpathways, respectively, and the entire pathways they belonged to were all found by the ICDS method (Table 1). Using the method ICDS-CNV method, we could not find any significant subpathway. If we consider the genetic differences or expression differences based on a single type of data, we may lose important information. However, ICDS exclusively identified 15 significant subpathways marked with red asterisks in Figure 2A, and the KEGG pathways they belong to could not be found based on the three other methods. Some pathways identified by ICDS were the chemokine signaling pathway and focal adhesion, which have been reported to be related to the occurrence and development of hepatocellular carcinoma (Zhao et al., 2011). It has been reported in the literature that the chemokine signaling pathway is involved in the establishment of a tumor-promoting microenvironment and in the development and progression of hepatobiliary cancer (Zlotnik and Yoshie, 2000). Drug targeting of the chemokine pathway is a promising approach for the treatment or even prevention of hepatobiliary cancer. Chemokines play a vital role in tumor progression and metastasis, where the binding of chemokines to their receptors leads to a conformational change, which activates signaling pathways and promotes migration (Zhao et al., 2011). Meanwhile, the subpathway path:04062_1 in the chemokine signaling pathway (Figure 2B), exclusively identified by ICDS, included the chemokine family (CC or CXC) and its receptors family (CCR or CXCR). All of these chemokine families exert their biological effects by binding to chemokine receptors that interact with G protein-linked transmembrane receptors (Decaillot et al., 2011). In the subpathway path:04062_1 (Figure 3A), the CXC motif chemokine 12 (CXCL12) is a chemokine protein that is differentially expressed between LIHC and normal samples (*p_gene_* = 1.53e-35), and both the expression of CCL20 and CCR2 are regulated by differential methylation (*p_methy_* = 3.07e-18, 2.3e-16). Importantly, the ICDS method not only recognized subregions of differential gene expressions but also detected some genetically or epigenetically diverse regions (e.g., CNVs and methylations). Another subpathway of the chemokine signaling pathway was path:04062_4, which contains 9 genes (Figure 3B). We found that four of these genes were mainly influenced by differential expressions and five were mainly influenced by differential methylation. Thus, our method can efficiently find dysfunctional local regions in biological pathways and indicate their perturbation by deriving specific types of molecular aberrations (CNV, differential methylations or differential gene expressions).

### Analyses of Head-Neck Squamous Cell Carcinoma Data

**FIGURE 4:**
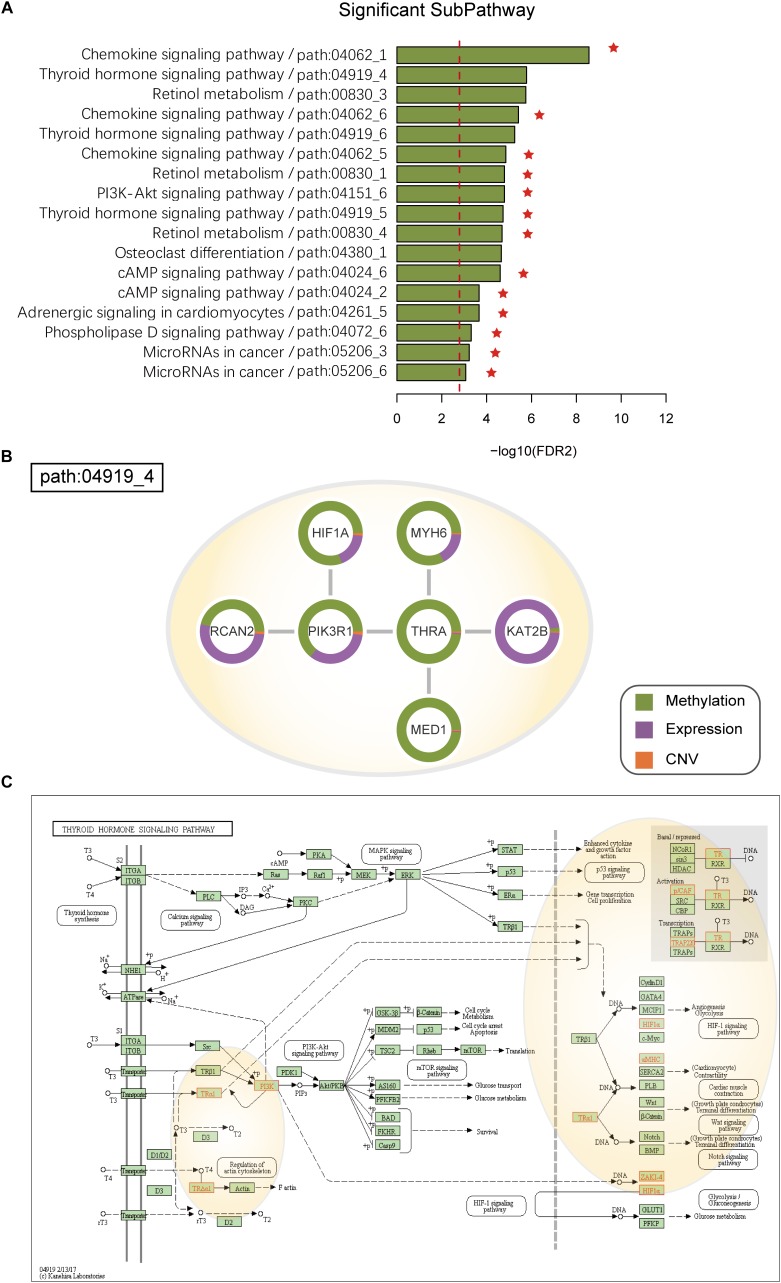
**(A)** SubPathways identified by ICDS with FDR < 0.001 in the HNSC dataset. The y-axis represents significant subpathways sorted by FDR2, while the x-axis represents the log-transformed FDR2. Compared to the three methods (ICDS-G, ICDS-CNV, and ICDS-M), the subpathways exclusively identified by ICDS method are marked with red asterisks. **(B)** Dysfunctional subpathway (path:04919_4) of thyroid hormone signaling pathway in HNSC. The vertex in the subnetwork represents a gene, and green and purple colors in the vertex represent the proportion of the gene’s differential expression scores and differential methylation scores between cancer samples and normal samples; orange colors represent the proportion of influence of CNV on gene expression. **(C)** Annotation of genes in path:04919_4 to the original thyroid hormone signaling pathway in KEGG. Genes are marked with red, and the light-yellow circle corresponds to path:04919_4.

The HNSC datasets were obtained from TCGA, and their detailed information is shown in Supplementary Table S1. ICDS identified 17 significant dysfunctional subpathways associated with HNSC belonging to 9 entire pathways and the subpathways exclusively identified by the ICDS method are marked with red asterisks in Figure 4A (Table 2), of which up to eight have been reported to be central to the growth and survival of cancer cells. Subpathways were selected that satisfied two tests with FDR1 < 0.001 and FDR2 < 0.001 (see Materials and Methods).

**Table 2:** Subpathways identified by ICDS with FDR <0.001 in the HNSC dataset.

SubpathID	Pathway	Size^∗^	FDR1	FDR2	ICDS-G	ICDS-CNV	ICDS-M
path:04062_1	Chemokine signaling pathway	41	<E-30	2.73E-09	√		
path:04919_4	Thyroid hormone signaling pathway	7	<E-30	1.67E-06			√
path:00830_3	Retinol metabolism	11	<E-30	1.79E-06			
path:04062_6	Chemokine signaling pathway	10	<E-30	3.82E-06	√		
path:04919_6	Thyroid hormone signaling pathway	5	<E-30	5.59E-06			
path:04062_5	Chemokine signaling pathway	8	<E-30	1.40E-05			
path:00830_1	Retinol metabolism	17	<E-30	1.60E-05			
path:04151_6	PI3K-Akt signaling pathway	10	<E-30	1.60E-05			
path:04919_5	Thyroid hormone signaling pathway	9	<E-30	1.86E-05			
path:00830_4	Retinol metabolism	7	<E-30	2.06E-05			
path:04380_1	Osteoclast differentiation	15	<E-30	2.21E-05			√
path:04024_6	cAMP signaling pathway	9	<E-30	2.48E-05			
path:04024_2	cAMP signaling pathway	11	<E-30	2.17E-04			
path:04261_5	Adrenergic signaling in cardiomyocytes	6	<E-30	2.20E-04			
path:04072_6	Phospholipase D signaling pathway	5	<E-30	4.90E-04			
path:05206_3	MicroRNAs in cancer	5	<E-30	6.0E-04			
path:05206_6	MicroRNAs in cancer	5	<E-30	8.50E-04			


*^∗^The number of genes in the subpathway.*

Path:04919_4 is a significant subpathways (Figure 4B and Supplementary Table S3) belonging to the thyroid hormone signaling pathway (Figure 4C). Many studies have confirmed that the thyroid hormone signaling pathway is a critical component in tumor progression (Kim and Cheng, 2013). Loss of normal function of thyroid-hormone receptors by deletion or mutation can contribute to cancer development, progression and metastasis. Thyroid Hormone Receptor Alpha (THRA) belongs to the nuclear receptor superfamily, is located on different chromosomes, and encodes thyroid hormone (T3) binding thyroid hormone receptor (TR) isoforms, which have been shown to mediate the biological activities of cells (Laudet et al., 1993; Wagner et al., 1995). TRs can function as tumor suppressors, because reduced expression of TRs due to hypermethylation or deletion of TR genes is found in human cancers. The samples had significantly different methylation of THRA (p_*methy*_ = 4.79e-12) in HNSC, and low expression of THRA is known to activate PIK3R1, which provides instructions for synthesizing a subunit of phosphatidylinositol 3-kinase (PI3K). PI3K signaling is important for many cell activities, including cell growth, division, and migration (Jaiswal et al., 2009). However, we calculated the *RS* of PIK3R1in HNSC, and its contributions with differential methylation were greater than that of differential expression (*p_methy_* = 4.78e-12; *p_gene_* = 1.46e-06) (Figure 4B).

Similarly, we compared the results of HNSC with the three methods above (ICDS-G, ICDS-CNV, and ICDS-M). Using the methods of ICDS-G and ICDS-M, we obtained two significant subpathways and the pathways they belonged to were also found by the ICDS method. However, 13 subpathways identified by ICDS were missing from all of the other methods (ICDS-G, ICDS-CNV, and ICDS-M) (Table 2). For example, the subpathway path:00830_3 in retinol metabolism pathway was identified by ICDS but not by ICDS-G, ICDS-CNV, or ICDS-M, and Supplementary Figures S3, S4 show the distribution of the activity score of path:00830_3, combined and individual data source, for the real subpathways and for the randomization cases. The local region of the subpathways was reported to be central to the growth and survival of cancer cells (Supplementary Figure S2A). Specifically, vitamin A (retinol) can control mucosal lesions before the occurrence of HNSC and prevent the occurrence of second primary tumors. Therefore, retinol metabolism is essential for the early diagnosis and treatment of HNSC. Retinoic acid (RA) is a critical signaling molecule that regulates gene transcription and the cell cycle (Tzimas and Nau, 2001), and retinal is then metabolized by NAD/NADP-dependent retinal dehydrogenases (RALDH) and by retinal oxidase enzymes to RA (Chen et al., 1995). Additionally, CYP26C1 in the path:00830_3 is involved in the metabolic breakdown of retinoic acid, which could be more effective in the growth inhibition of cancer cells (Thatcher and Isoherranen, 2009). Moreover, in the HNSC dataset, some genes mainly showed differences in the degree of methylation compared to normal samples, such as CYP26C1 (*p_methy_* = 9.25e-34) and ALDH1A2 (*p_methy_* = 1.65e-13). Other components in the same subpathway, path: 00830_3, mainly showed differences in the degree of expression compared to normal samples, such as AOX1 (*p_gene_* = 3.11e-18) and ADH4 (*p_gene_* = 2.75e-38) (Supplementary Figure S2B). Therefore, the ICDS method that we proposed can effectively identify disordered genetic and epigenetic subpathways.

### Analyses of Cervical Squamous Cell Carcinoma and Endocervical Adenocarcinoma Data

We applied ICDS to identify dysfunctional subpathways in CESC (see Materials and Methods). With the threshold of FDR1 < 0.001, we obtained four significant subpathways that had just exceeded the threshold FDR2 (Supplementary Table S4), and all of these subpathways were associated with the development and progression of CESC tumors. Meanwhile, using the method of ICDS-G, we obtained three significant subpathways, and the pathways they belonged to were also found by the ICDS method (Supplementary Tables S4, S5). Subpathway 04020_1 in the calcium-signaling pathway, identified by ICDS, was simultaneously neglected by the other three methods.

**FIGURE 5:**
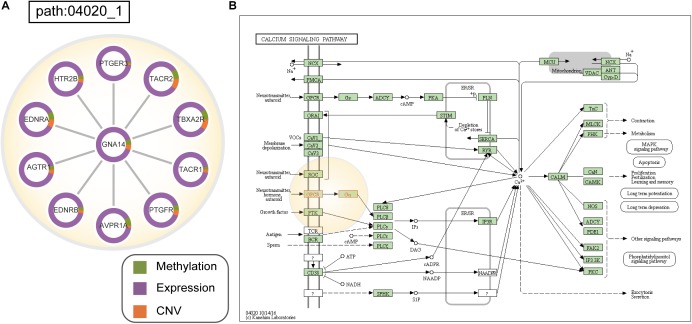
**(A)** Dysfunctional subpathway (path:04020_1) of calcium signaling pathway in CESC. The vertex in the subnetwork represents a gene, and green and purple colors in the vertex represent the proportion of the gene’s differential expression scores and differential methylation scores between cancer samples and normal samples; orange colors represent the proportion of the influence of CNV on gene expression. **(B)** Annotation of genes in path:04020_1 to the original calcium signaling pathway in KEGG. Genes are marked with red, and the light-yellow circle corresponds to path:04020_1.

Interestingly, subpathway 04020_1 (Figure 5A) in the calcium-signaling pathway is involved many G-protein coupled receptors (GPCRs), such as TACR1, TACR2, and HTR2B, and downstream heterotrimeric guanine nucleotide-binding proteins (G-proteins; GNA14) (Figure 5B). In this subpathway, many GPCRs had significant patterns of expression changes in CESC patients, such as TACR1 (*p_gene_* = 9.92e-32), TACR2 (*p_gene_* = 3.82e-08), and HTR2B (*p_gene_* = 2.76e-26). Moreover, with CESC samples, AVPR1A, which is a GPCR in cells, mainly showed differences in methylation and expression compared to normal samples. Many studies have shown that the abnormal expression and activity of GPCRs is associated with the development and progression of cancers (Audigier et al., 2013; Moody et al., 2016). GPCRs play a role as key transducers of signals from the extracellular milieu to the intracellular milieu of cells. It has been confirmed that many GPCRs are highly expressed in specific cancer cells, such as in cervical, breast, and prostate cancer cells (Dey et al., 2010). Similarly, abnormal expression of GPCRs contributes to the development of cancer (Radhika and Dhanasekaran, 2001; O’Hayre et al., 2013). Furthermore, initial signal transduction, such as that of calcium signaling, is achieved primarily by GPCRs activated downstream of heterotrimeric G proteins (Hanlon and Andrew, 2015; Schafer and Blaxall, 2017). Calcium-signaling channels are important for the proliferation, migration, and differentiation of cells, including tumors. CESC is associated with the significant upregulation of calcium-signaling pathways (Perez-Plasencia et al., 2007; Monteith et al., 2012).

### Comparison of ICDS With Other Pathway Analysis Methods

In recent years, the pathway-based and subpathway-base approaches have become the first choice for complex disease analysis in order to yield biological insight. To explore whether ICDS could provide new biological insights in identifying important subpathways, we compared ICDS with three widely used pathway-based and subpathway-base approaches including SPIA (Tarca et al., 2009), GSEA (Subramanian et al., 2005), and SubpathwayMiner (Li et al., 2009). These three methods mainly identify dysregulated pathways or subpathways by using gene expression data, however, the ICDS method identifies the dysregulated subpathways by integrating the three types of data: DNA methylation, CNV, and gene expression. In order to compare the results of the above methods uniformly, we chose to compare the entire pathways identified by them. In HNSC datasets, ICDS identified 17 statistically significant subpathways, which belong to nine entire pathways. SPIA and GSEA found five and eight significant pathways, and SubpathwayMiner did not yield any significant pathways. Through comparing the results of these methods, we found that ICDS identified six statistically significant pathways, which were simultaneously missed by other methods (Supplementary Table S6). The significant pathways exclusively identified by ICDS, such as the cAMP signaling pathway, chemokine signaling pathway, Retinol metabolism etc., have been well reported to be associated with the development of HNSC (Tzimas and Nau, 2001; Tanaka et al., 2005). For example, the thyroid hormone signaling pathway and retinol metabolism were reported to be central to the growth and survival of cancer cells. A subpathway of Retinol metabolism identified by ICDS methods (Supplementary Figure S2A) is essential for the early diagnosis and treatment of HNSC. These results indicate that the ICDS method may uncover something new dysregulated subpathways.

## Discussion

The occurrence and development of diseases, especially cancer, involves a complex biological network (Zou et al., 2016). Genetic variation, epigenetic changes, abnormal gene-expression levels, and many other factors will change in the characteristics of living organisms. With the generation of large-scale sequencing data, more opportunities exist to study the multi-omics molecular mechanisms of cancer development. In systems biology, dysfunctional genes may jointly activate biological pathways. Therefore, the most critical step in exploring complex disease mechanisms is to identify the functional pathways in which these dysregulated genes are located. We proposed the concept of subpathways in our previous work (Li et al., 2009, 2013). The subpathway, defined as a local region of an entire pathway, contains fewer components, which enables a more subtle and accurate interpretation of the biological function of disturbances involved in cancer progression.

In this study, the employed method was based on *a priori* biological pathways (e.g., KEGG), each of which represents a network of interactions between genes, proteins, and chemical molecules. The main purpose of this study was to discover important dysfunctional subregions based on the pathway topological structure. ICDS used Fisher’s combined probability test to integrate gene expression, CNVs, and methylation to calculate the *RS* of genes. As the gene expression, CNV and DNA methylation are not completely independent, and thus the independence assumption of Fisher’s combined probability test may be violated here. This may be a limitation of our ICDS method. Alternatively, the Brown’s method (Poole et al., 2016) can also be used to integrate multiple data source, and it does not suffer from this limitation. A larger *RS* in cancer indicated a greater correlation between the gene and the cancer phenotype. Next, we used a greedy algorithm to search for subpathways in each KEGG pathway network, so that subpathway activities were local maxima. This algorithm have also been used to identified subnetwork markers of breast cancer metastasis in the human protein–protein interaction network previously, and achieved higher accuracy in the classification of metastatic versus non-metastatic tumors (Chuang et al., 2007). To avoid excessive redundancy in the candidate subpathways, we set several parameters, such as seed gene (*p-*value of combined statistic S < 0.001), subpathway size (5 < size < 100), and overlap between subpathways (i.e., Jaccard index between each pair of subpathways in the same pathway < 0.6), which can be set by a user of the ICDS package.

We applied the ICDS method to LIHC, HNSC, and CESC datasets. Based on these analyses, we demonstrated that ICDS can effectively identify dysfunctional subpathways correlated with a cancer phenotype. For the HNSC dataset, the subpathway path:04062_1 was the most significant subpathway and included 41 genes belonging to chemokine signaling pathway. Studies have confirmed that the chemokine signaling pathway is a critical component of tumor progression. These genes did not simultaneously have changes in copy number, methylation, and gene expression. However, these subregions could still be found through our integration algorithm, which is the most prominent advantage of our method. To further validate the ICDS method, we compared it with three other methods that only considered one type of data – gene expression, CNV, or DNA methylation – named as ICDS-G, ICDS-CNV, and ICDS-M, respectively. The results showed that the ICDS method was able to identify new risk subpathways associated with cancer that were simultaneously neglected by the other three methods. Thus, it is essential to integrate multi-omics data to identify additional dysfunctional subpathways in cancer. In the future, we will involve other omics data, such as proteomics, to improve our ICDS method.

To provide users with convenient and simple analytical tools, we have integrated the ICDS, ICDS-G, ICDS-CNV, and ICDS-M methods into an available R-based package on CRAN^3^. If users are considering using the ICDS method, they need to input three datasets of gene expression, copy number, and methylation. The ICDS-package will produce a prioritized list of subpathways. With this method, ICDS is used to find key subpathways related to cancer phenotypes, and it is expected that it can be used to mine for key subnetworks within some prior networks (e.g., the PPI network) based on integrating DNA methylation, CNV, and gene expression data. In addition, ICDS may identify key subpathways as biomarkers to distinguish high and low risk cancer patients. For this purpose, researchers should input the molecular profile of genes with different stage samples, such as patients in different stages of glioma. Therefore, we have developed a free and robust tool to identify dysfunctional subpathways in cancer by integrated multi-omics data.

## Author Contributions

JH, YZ, and LC conceived and designed the study. SL and BZ developed the software. YY analyzed the data and implemented the methodology. YJ revised the manuscript. YZ provided constructive discussions. JH and LC drafted the manuscript. All the authors read and agreed to the manuscript.

## Conflict of Interest Statement

The authors declare that the research was conducted in the absence of any commercial or financial relationships that could be construed as a potential conflict of interest.

## Footnotes

1 https://tcga-data.nci.nih.gov/tcga/

2 https://cran.r-project.org/web/packages/ICDS

3 https://cran.r-project.org/web/packages/ICDS
