# Supplementary material for: Identification of Cancer Dysfunctional Subpathways by Integrating DNA Methylation, Copy Number Variation, and Gene-Expression Data
Source: Front Genet. 2019 May 15;10:441. doi: 10.3389/fgene.2019.00441 (PMC6529853; doi:10.3389/fgene.2019.00441)
Supplement: Supplementary file 1 [file Data_Sheet_1.docx]

Supplementary Material

Identification of cancer dysfunctional subpathway by integrating DNA methylation, copy number variation, and gene-expression data

Siyao Liu^1,†^, Baotong Zheng^1,†^, Yuqi Sheng^1,†^, Qingfei Kong^2^, Ying Jiang^3^, Yang Yang^1^, Xudong Han^2^, Liang Cheng^1,^*, Yunpeng Zhang^1,^*, and Junwei Han^1,^*

**Inventory of Supplementary Information**

1. Supplementary Table S1-S6

2. Supplementary Figure S1-S4

**Supplementary Table S1. Summary of detailed data information.**

| **Datasets** | **LIHC** | **HNSC** | **CESC** |
| --- | --- | --- | --- |
| **Expression profile** |  |  |  |
| **No. of samples** | 424 | 546 | 309 |
| **Tumor** | 374 | 502 | 306 |
| **Normal** | 50 | 44 | 3 |
| **No. of genes** | 19754 | 19754 | 19754 |
| **DNA Methylation profile** |  |  |  |
| **No. of samples** | 429 | 580 | 312 |
| **Tumor** | 379 | 530 | 309 |
| **Normal** | 50 | 50 | 3 |
| **No. of genes** | 20105 | 20114 | 20108 |
| **Copy Number Variation** |  |  |  |
| **No. of samples** | 370 | 522 | 295 |
| **No. of genes** | 24776 | 24776 | 24776 |

**Supplementary Table S2. Detailed subpathway information identified in the LIHC dataset**

| **SubpathID** | **pathway** | **gene** |
| --- | --- | --- |
| **path00230_1** | **Purine metabolism** | **GMPR2, ENTPD8, RRM2, NTPCR, ADCY2, POLR3F, GUK1, GMPS, POLR1A, ADCY10, ZNRD1, RRM1, POLR3K, POLR3H, POLR3E, POLR3D, POLR3C, POLR3A, POLR2K, POLR2J3, POLR2H, POLR2G, POLR2D, POLR1D, POLR1C, PNPT1, PDE7A, PDE6H, PDE6G, PDE6D, PDE6C, PDE6B, PDE5A, PDE3B, PDE2A, PDE1A, PDE10A, NUDT2, NT5M, NME7, PRIM2, NME6, NME3, IMPDH2, HDDC3, GUCY2C, FHIT, ENTPD6, ENTPD4, ENTPD1, ENPP4, DCK, CANT1, ATIC, APRT, AMPD1, ADSS, ADSL, ADK, ADCY6, ADCY3** |
| **path00240_1** | **Pyrimidine metabolism** | **DTYMK, DUT, RRM2, TYMS, ENTPD8, PRIM2, UCKL1, UMPS, CANT1, ZNRD1, POLR3F, UCK2, UCK1, TXNRD2, TK1, RRM1, PRIM1, POLR3K, POLR3H, POLR3E, POLR3D, POLR3C, POLR3A, POLR2K, POLR2J3, POLR2H, POLR2G, POLR2D, POLR1D, POLR1C, POLR1A, POLE3, POLE2, POLD4, POLD3, POLD1, POLA2, POLA1, NUDT2, NT5M, NME7, NME6, NME3, NME1-NME2, NME1, ITPA, ENTPD6, ENTPD4, DCTPP1, CTPS2, CMPK1** |
| **path04380_1** | **Osteoclast differentiation** | **LILRA4, LILRA2, LILRA1, LILRB5, LILRA3, LILRA5, LILRB3, FCGR1A, LILRB4, LILRB2, LILRB1, LILRA6, FCGR3B** |
| **path00830_1** | **Retinol metabolism** | **UGT2B7, CYP2C8, CYP4A11, CYP26A1, LRAT, ADH4, ADH1A, UGT2B10, UGT2A1, SDR16C5, RPE65, RDH8, CYP2B6, RDH5, RDH16, HSD17B6, CYP2C9, CYP26C1, CYP1A2, ADH6, ADH5, ADH1C, ADH1B** |
| **path04062_1** | **Chemokine signaling pathway** | **GRK1, CCR2, CCL23, CXCL12, CCL11, CCL20, CCL7, ADRBK1, XCR1, GRK4, CXCR5, CXCR2, CXCR1, CXCL14, CXCL13, CX3CR1, CCR5, CCR3, CCL8, CCL4, CCL26, CCL18, CCL13, CCL1** |
| **path04510_10** | **Focal adhesion** | **PXN, CRK, PTK2, ITGA6, COL6A6, COL5A1, COL6A3, CRKL** |
| **path04152_1** | **AMPK signaling pathway** | **PRKAB1, ADRA1A, GYS2, PRKAG1, RPTOR, RHEB, CRTC2, ACACA, TSC2, TSC1, STRADA, STK11, RPS6KB2, PRKAG3, PRKAB2, PFKFB2, PCK1, MTOR, MLYCD, ELAVL1, EEF2K, CD36, CAB39, AKT1S** |
| **path05166_1** | **HTLV-I infection** | **ANAPC4, PTTG1, CDC16, MAD2L1, ANAPC2, BUB3, CDC20, ANAPC1, CDC27, CDC26, CDC23, CCNB2, BUB1B, ANAPC7, ANAPC5, ANAPC11** |
| **path04062_4** | **Chemokine signaling pathway** | **GNAI1, ADCY2, CCR2, CCL23, CXCL12, CCL11, CCL20, GRK6, CCL14** |
| **path00240_3** | **Pyrimidine metabolism** | **ENTPD1, PRIM2, UCKL1, RRM2, UMPS, DTYMK, DUT** |
| **path04062_7** | **Chemokine signaling pathway** | **ADCY6, GNAI1, ADCY2, CCR2, CCL23, CXCL12, CCL11, CCL20, ADCY3, CCL7** |
| **path04110_10** | **Cell cycle** | **CCNA2, CDK1, CDC16, PTTG1, MAD2L1, ANAPC2, BUB3, ANAPC4** |
| **path04110_11** | **Cell cycle** | **SFN, CCNB1, CDC16, PTTG1, MAD2L1, ANAPC2, BUB3, GADD45B, ANAPC4** |
| **path04630_4** | **Jak-STAT signaling pathway** | **IFNA7, LIFR, GHR, TSLP, IFNA8** |
| **path00240_2** | **Pyrimidine metabolism** | **DHODH, UMPS, UCKL1, ENTPD8, PRIM2, RRM2, DTYMK** |
| **path00240_4** | **Pyrimidine metabolism** | **DCK, DCTPP1, PRIM2, DUT, RRM2, DTYMK, TYMS, ENTPD8** |
| **path00230_4** | **Purine metabolism** | **PAICS, ADSL, ENTPD8, RRM2, NTPCR, ADCY2, POLR3F, GUK1, GMPS, POLR1A** |
| **path04110_1** | **Cell cycle** | **ANAPC4, PTTG1, CDC16, MAD2L1, ANAPC2, BUB3, CDC20, ANAPC1, RB1, CDK1, PKMYT1, FZR1, CDK7, CDC27, CDC26, CDC23, CCNB2, GADD45B, CCNB1, BUB1B, BUB1, ANAPC7, ANAPC5, ANAPC13, ANAPC11** |
| **path04114_1** | **Oocyte meiosis** | **CDK2, CDC16, PTTG1, MAD2L1, ANAPC2, ANAPC4, CDC20, ANAPC1, PPP3R2, PPP3CB, PPP3CA, ANAPC7, FBXO43, ESPL1, CDC27, CDC26, CDC23, CCNB2, CPEB3, CCNB1, CDK1, CALML5, CALM3, CALM2, BUB1, ANAPC5, ANAPC13, ANAPC11** |

**Supplementary Table S3. Detailed subpathway information identified in the HNSC dataset**

| **SubpathID** | **pathway** | **Gene** |
| --- | --- | --- |
| **path04062_1** | **Chemokine signaling pathway** | **CXCR1, CCL14, CXCR2, CCR8, CCL3, CCL23, CCR2, ADCY2, GNAI1, GNB1, XCR1, JAK2, GRK4, GRK1, GNAI2, GNGT1, CXCL6, CXCL5, CXCL12, CX3CR1, CCR7, CCR6, CCR5, CCR3, CCR1, CCL8, CCL7, CCL5, CCL4, CCL28, CCL26, CCL25, CCL24, CCL2, CCL18, CCL16, CCL15, CCL13, CCL11, CCL1, ADCY8** |
| **path04919_4** | **Thyroid hormone signaling pathway** | **RCAN2, PIK3R1, THRA, MED1, KAT2B, MYH6, HIF1A** |
| **path00830_3** | **Retinol metabolism** | **LRAT, ADH4, ADH1B, RDH5, AOX1, CYP2C9, CYP26C1, CYP2C18, ALDH1A2, CYP3A4, ADH5** |
| **path04062_6** | **Chemokine signaling pathway** | **ADCY3, GNAI1, CXCR2, CCL14, GNB1, CCR8, CCL3, CCL23, CCR2, GNGT1** |
| **path04919_6** | **Thyroid hormone signaling pathway** | **MED4, MED1, THRA, KAT2B, MYH6** |
| **path04062_5** | **Chemokine signaling pathway** | **STAT2, JAK2, CXCR2, CCL14, CCR8, CCL3, CCL23, CCR2** |
| **path00830_1** | **Retinol metabolism** | **CYP4A11, CYP2C9, CYP26C1, CYP2C18, CYP3A4, CYP3A5, CYP2B6, AOX1, ADH4, ADH1B, RDH5, ADH1A, UGT2B11, RETSAT, HSD17B6, DHRS9, CYP1A2** |
| **path04151_6** | **PI3K-Akt signaling pathway** | **HSP90AA1, HSP90B1, AKT1, PPP2R1B, PIK3R6, GNB1, TCL1B, CREB3L3, GNGT1, CDC37** |
| **path04919_5** | **Thyroid hormone signaling pathway** | **SRC, PIK3R1, THRA, MED1, KAT2B, MYH6, PLN, HIF1A, PIK3R2** |
| **path00830_4** | **Retinol metabolism** | **CYP26A1, ADH4, ADH1B, CYP2C9, CYP26C1, CYP2C18, CYP3A4** |
| **path04380_1** | **Osteoclast differentiation** | **LILRB1, LILRA2, LILRA1, LILRB4, LILRA3, LILRA4, FCGR1A, TNFRSF11A, FCGR3A, LILRB5, LILRB3, LILRB2, LILRA6, FCGR3B, FCGR2A** |
| **path04024_6** | **cAMP signaling pathway** | **GNAI1, SSTR1, HTR1B, GHSR, HTR1A, NPY, ADCY2, ADCY8, GNAI2** |
| **path04024_2** | **cAMP signaling pathway** | **SSTR1, NPY, HTR1B, GHSR, HTR1A, NPY1R, CHRM1, PTGER3, HTR1F, HTR1D, FFAR2** |
| **path04261_5** | **Adrenergic signaling in cardiomyocytes** | **CAMK2A, SCN7A, CACNG5, PPP2R1B, CACNG8, CAMK2B** |
| **path04072_6** | **Phospholipase D signaling pathway** | **GNA13, CXCR2, PIK3R6, CYTH4, AVPR1A** |
| **path05206_3** | **MicroRNAs in cancer** | **GRB2, MIR129-2, MIR10B, HOXD10, MIR10A** |
| **path05206_6** | **MicroRNAs in cancer** | **PRKCG, MIR129-2, MIR10B, HOXD10, MIR30E** |

**Supplementary Table S4. Subpathways identified by ICDSwith fdr<0.001 in the CESC dataset.**

| **SubpathID** | **pathway** | **Size of subpathway** | **AS** | **FDR1** | **ICDS-G** | **ICDS-CNV** | **ICDS-M** |
| --- | --- | --- | --- | --- | --- | --- | --- |
| **path04020_1** | **Calcium signaling pathway** | **11** | **30.29** | **<E-15** |  |  |  |
| **path04022_1** | **cGMP-PKG signaling pathway** | **15** | **48.33** | **<E-15** | $\boldsymbol{\surd}$ |  |  |
| **path04270_1** | **Vascular smooth muscle contraction** | **12** | **39.76** | **<E-15** | $\boldsymbol{\surd}$ |  |  |
| **path04270_2** | **Vascular smooth muscle contraction** | **9** | **41.86** | **<E-15** | $\boldsymbol{\surd}$ |  |  |

**SupplementaryTable S5. Detailed subpathway information identified in the CESC dataset**

| **SubpathID** | **pathway** | **Gene** |
| --- | --- | --- |
| **path04020_1** | **Calcium signaling pathway** | **PTGFR, GNA14, PTGER3, AVPR1A, TACR1, EDNRB, HTR2B, AGTR1, TBXA2R, TACR2, EDNRA** |
| **path04022_1** | **cGMP-PKG signaling pathway** | **GUCY1A2, PDE2A, PRKG1, KCNMB1, PLN, KCNMA1, CACNA1C, MRVI1, ATP1B2, SLC8A1, NPR2, NOS3, KCNMB2, ITPR1, ATP2B4** |
| **path04270_1** | **Vascular smooth muscle contraction** | **PPP1CB, MYL9, ACTA2, MYH11, ACTG2, PPP1R12B, PPP1R14A, MYLK, ROCK2, PPP1R12C, CALM2, CALM1** |
| **path04270_2** | **Vascular smooth muscle contraction** | **MRVI1, PRKG1, KCNMB1, KCNMA1, PPP1R12B, MYL9, GUCY1A2, KCNMB2, ITPR1** |

**Supplementary Table S6. Dysfunctional pathways identified by ICDS and other methods (SPIA, GSEA, SubpathwayMiner) in the HNSC dataset**

| Pathway names | ICDS | SPIA | SubpathwayMiner | GSEA |
| --- | --- | --- | --- | --- |
| Adrenergic signaling in cardiomyocytes | √ |  |  | √ |
| Alcoholism |  |  |  | √ |
| cAMP signaling pathway | √ |  |  |  |
| Cell cycle |  |  |  | √ |
| Chemokine signaling pathway | √ |  |  |  |
| ECM-receptor interaction |  | √ |  |  |
| Herpes simplex infection |  | √ |  |  |
| MicroRNAs in cancer | √ |  |  | √ |
| Neuroactive ligand-receptor interaction |  |  |  | √ |
| Osteoclast differentiation | √ |  |  |  |
| Phospholipase D signaling pathway | √ |  |  |  |
| PI3K-Akt signaling pathway | √ |  |  | √ |
| Retinol metabolism | √ |  |  |  |
| Small cell lung cancer |  | √ |  |  |
| Systemic lupus erythematosus |  | √ |  | √ |
| Thyroid hormone signaling pathway | √ |  |  |  |
| Viral carcinogenesis |  | √ |  | √ |


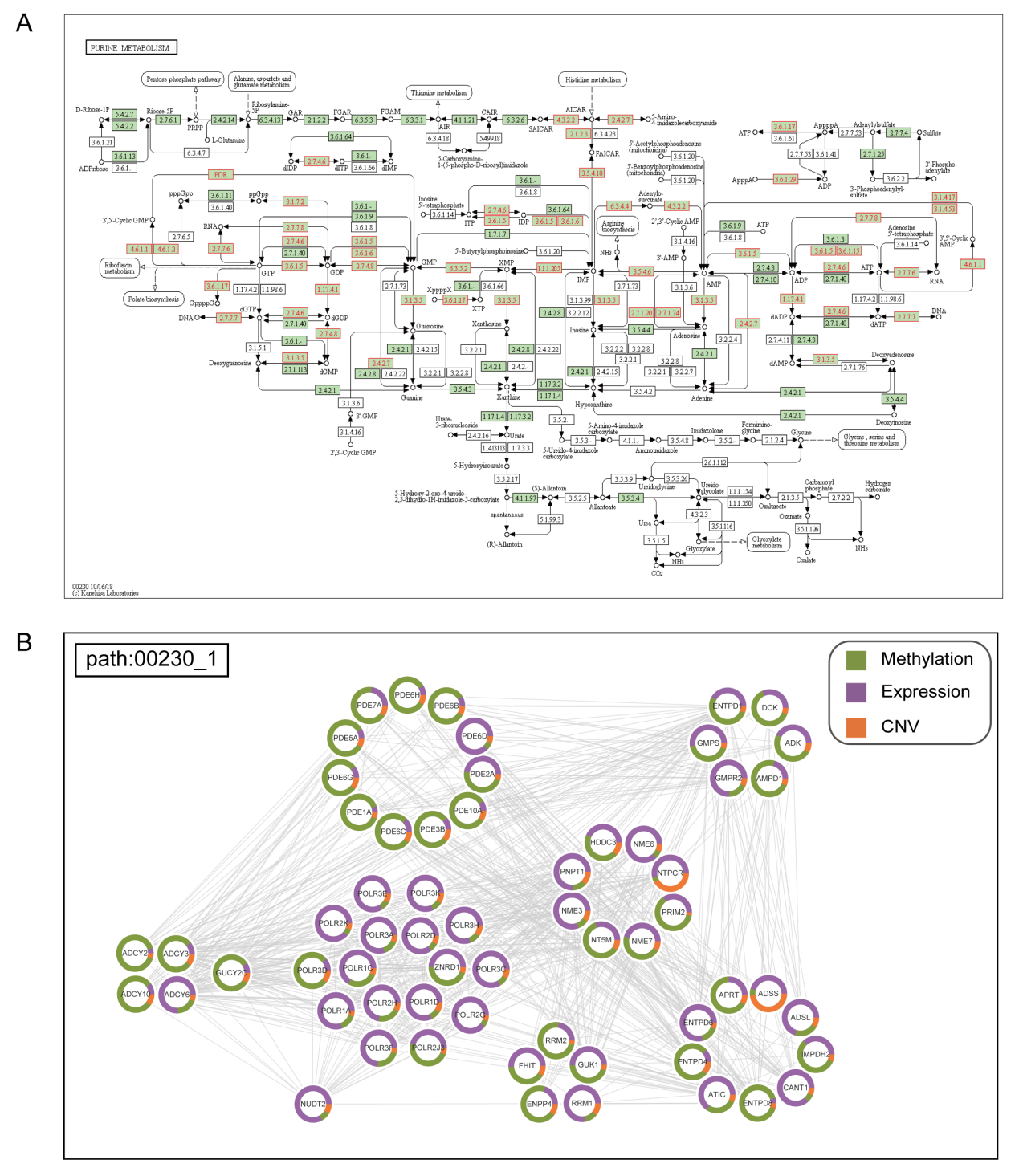


**Supplementary Figure S1.** (A) Annotation of genes in thepath:00230_1 to the original purine metabolism pathway in KEGG. Genes are marked with red.(B) Subnetwork of path:00230_1.The vertex in the subnetwork represents a gene, in which green andpurplecolours in the vertex represent the proportion of gene’s different expression score and different methylation score between cancer samples and normal and orange colours represent the proportion of influence of copy number variation on gene expression.


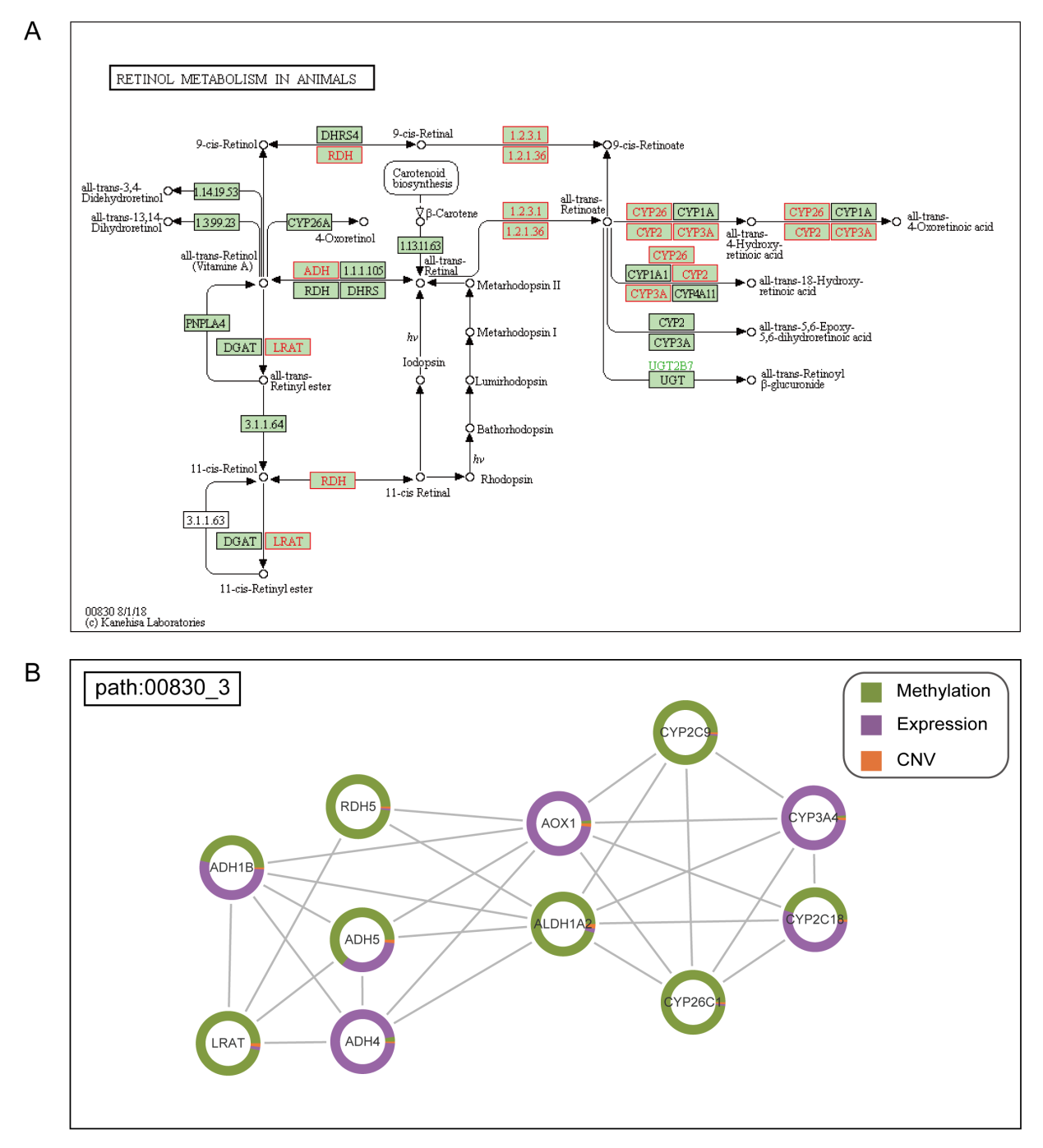


**Supplementary Figure S2.** (A) Annotation of genes in thepath:00830_3 to the original retinol metabolism in animals pathway in KEGG. Genes are marked with red. (B) Subnetwork of path:00830_3. The vertex in the subnetwork represents a gene, in which green andpurplecolours in the vertex represent the proportion of gene’s different expression score and different methylation score between cancer samples and normal and orange colours represent the proportion of influence of copy number variation on gene expression.


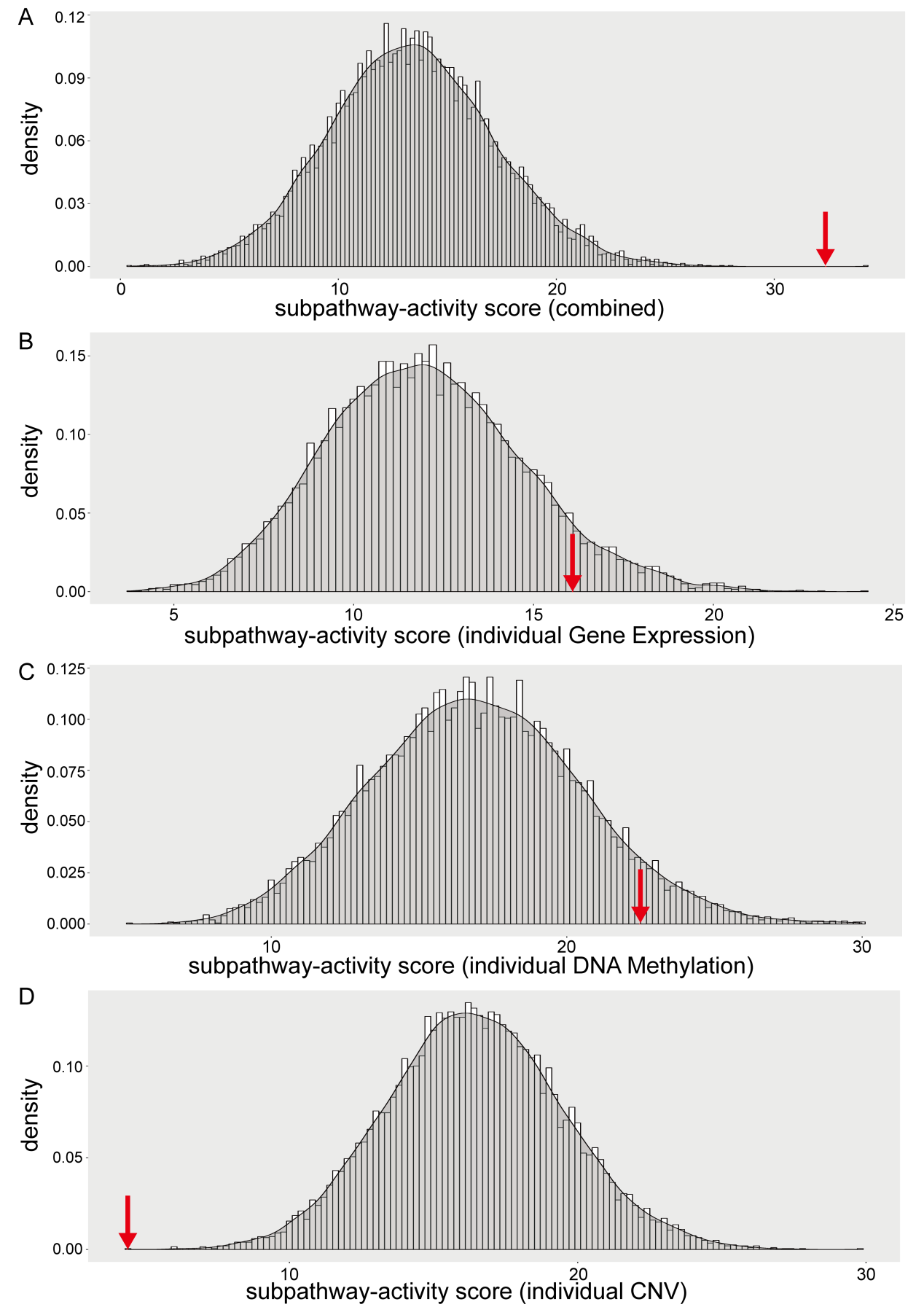


**Supplementary Figure S3.** Distribution of subpathway activity scores (AS) of individual and combined data sources for the first randomization strategy. Red arrow represents the indexes of the real AS of path:00830_3 in the distribution. (A) Distribution of random AS in the combined datasets of HNSC; (B) Distribution of random AS in the Gene Expression dataset of HNSC; (C) Distribution of random AS in the DNA Methylation dataset of HNSC; (D) Distribution of random AS in CNV dataset of HNSC.


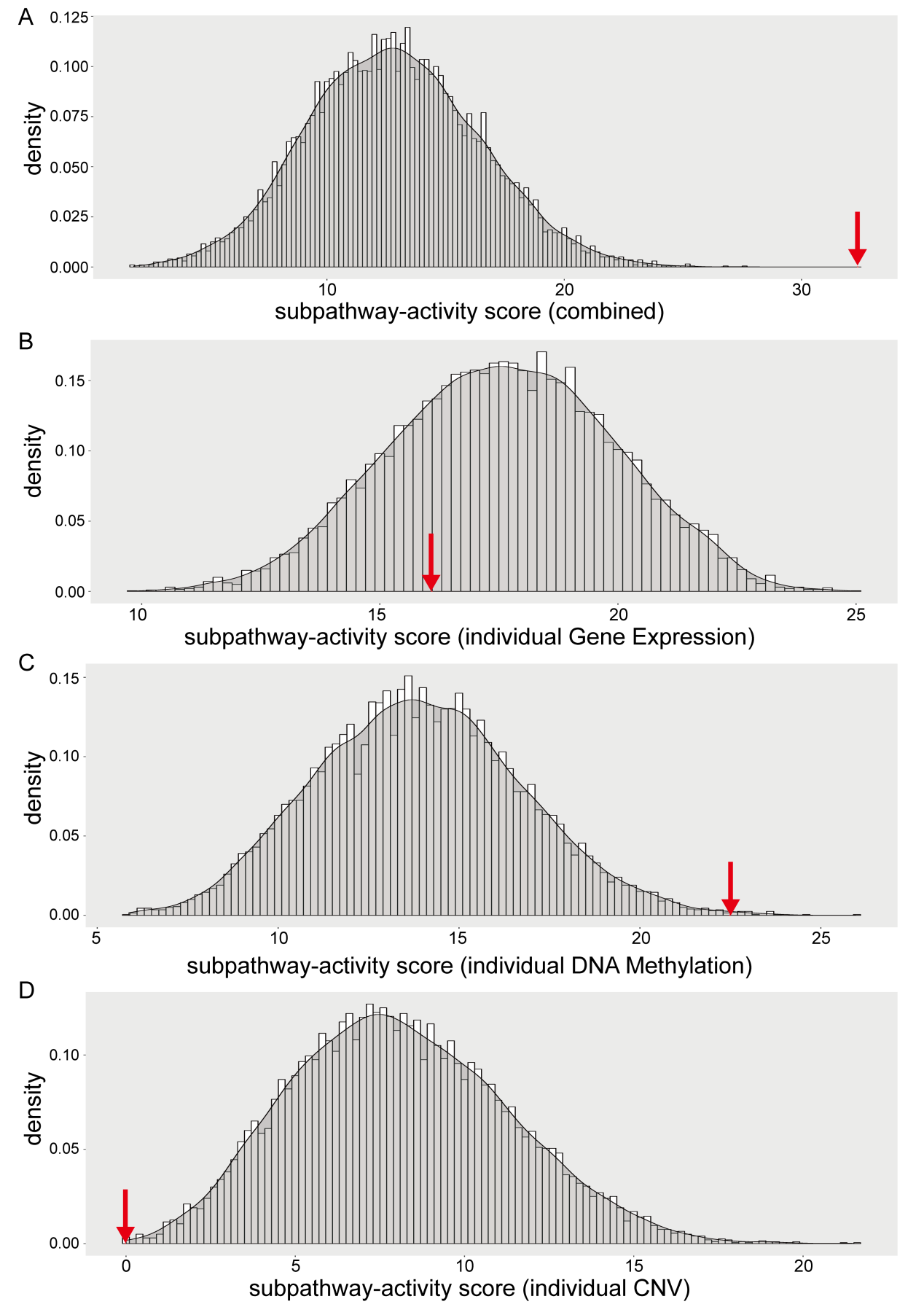


**Supplementary Figure S4.** Distribution of subpathway activity scores (AS) of individual and combined data sources for the second randomization strategy. Red arrow represents the indexes of the real AS of path:00830_3 in the distribution. (A) Distribution of random AS in the combined datasets of HNSC; (B) Distribution of random AS in the Gene Expression dataset of HNSC; (C) Distribution of random AS in the DNA Methylation dataset of HNSC; (D) Distribution of random AS in CNV dataset of HNSC.
